# Nicotinamide riboside supplementation restores microglial health and improves cognition in aged male mice

**DOI:** 10.1007/s11357-025-01959-1

**Published:** 2025-11-22

**Authors:** Ramkumar Thiyagarajan, Rupadevi Muthaiah, Bhavana Sreevelu, Owen P. Treanor, Yonas Redae, Reem Berman, Anna L. Davis, Nanda Yellapu, Spencer R. Rosario, Lee D. Chaves, Kenneth L. Seldeen, Bruce R. Troen

**Affiliations:** 1 Division of Geriatrics, Department of Internal Medicine, Landon Center On Aging, University of Kansas Medical Center, Kansas City, KS USA; 2 ICMR-Vector Control Research Center, Indira Nagar Medical Complex, Puducherry, India; 3 Department of Biostatistics and Bioinformatics and Experimental Therapeutics, Roswell Park Comprehensive Cancer Center, Buffalo, NY USA; 4 Research Service, Veterans Affairs Kansas City Healthcare System, Kansas City, MO USA

**Keywords:** Aging, Cognition decline, Microglia, Disease-associated microglia, NAD metabolism

## Abstract

Cognitive impairment affects 1 in 6 individuals over 60, with over 75 million projected by 2030. Age-related changes in microglial function and declining nicotinamide adenine dinucleotide (NAD^+^) levels may contribute to cognitive decline. Although nicotinamide riboside (NR) supplementation can restore NAD^+^ levels in aged mice, its effects on microglial phenotype and cognition during normal aging remain unclear. We assessed cognitive function, neuroinflammation, and microglial gene expression in 6-month (Young) and 22-month (Aged) mice, along with aged mice supplemented with NR (Aged + NR; 400 mg/kg body weight) for 8 weeks. Aged mice exhibited impaired cognition and increased gene expression related to neuroinflammation. NR supplementation improved or prevented the decline in nest-building ability, Y-maze spontaneous alternation, and novel object recognition, which are reflective of instrumental activities of daily living, spatial working memory, and recognition memory. NR supplementation diminished microglial (IBA1) and astrocytic (GFAP) activation, resembling the young phenotype. Gene expression profiling revealed reduced microglial activation, inflammatory pathways, and chemokine production in Aged + NR mice, along with upregulation of genes associated with learning, memory, and gliogenesis. NR lowered transcriptional signatures from age-dependent (ADEM) and disease-associated (DAM) microglia and enhanced homeostatic state profiles. Metabolic pathway analysis of microglial transcripts indicated that NR suppressed age-induced increases in fatty acid metabolism. This was supported by immunostaining, which showed reduced lipoprotein lipase (LPL), a DAM marker, in the cortex and hippocampus. Overall, NR appeared to mitigate age-related cognitive decline by shifting microglial gene expression and metabolism toward a younger phenotype, suggesting potential therapeutic relevance for healthy brain aging.

**Supplementary Information:**

The online version contains supplementary material available at https://doi.org/10.1007/s11357-025-01959-1.

## Introduction

Age-related chronic inflammation significantly disrupts cellular metabolism and promotes a shift toward a pro-inflammatory state within cells [1, 2]. These changes progressively impair physiological function and increase vulnerability to age-related diseases [2], contributing to the poor quality of life in older individuals [1, 3]. Among these conditions, cognitive decline is particularly concerning, beginning with subtle impairments in memory, attention, and executive function [4, 5]. Age is significantly associated with progression from cognitively intact to mild cognitive impairment [6] and eventually to dementia, severely hindering independence and quality of life. Dementia is increasingly widespread with age, affecting 13.2% of people aged 75 to 84, and 33.4% of people aged 85 or older [7]. The rising prevalence and the economic burden of caregiving and medical care highlight the importance of understanding the cellular and molecular basis of cognitive decline with age [7].

In response to a chronic inflammatory environment, microglial cells, the resident brain macrophages, lose resiliency through loss of transcriptional control that reduces the ability to sense and respond to surrounding signals [8]. Microglia respond to injury, pathogens, and inflammatory signals, remove cellular debris, and participate in synaptic pruning, a process critical to neural plasticity and cognitive health [9]. However, due to increasing inflammation during aging, these cells undergo significant changes in function and show disease-associated phenotypes, and their dysfunction coincides with cognitive decline [10–12]. Previous studies have shown that microglial activation or dysfunction is pivotal in the pathophysiology of age-related cognitive decline, including Alzheimer’s disease (AD) [13–15]. This highlights the critical importance of maintaining microglial health in mitigating cognitive decline as we age.

Age-related cognitive decline is also associated with metabolic dysregulation [16, 17] — in particular, lower levels of the enzyme cofactor NAD^+^ [18, 19]. Inflammaging and oxidative stress deregulate NAD^+^ metabolism by activating NAD + consuming enzymes (e.g., sirtuins, PARPs, and CD38) or inhibiting NAD^+^ generating enzymes like NAMPT, thus leading to a decline in NAD^+^ [20]. NAD^+^ is a critical co-factor for cellular metabolism, and decreases in NAD + contribute to age-related declines in cellular bioenergetics, genomic stability, mitochondrial homeostasis, and cell survival [21]. Supplementation with NAD^+^ precursors, such as nicotinamide riboside (NR) and nicotinamide mononucleotide (NMN), restores NAD^+^ levels in aging tissues [22–24] and the brain [25]. In the brain, replenishing NAD^+^ levels has been shown to enhance mitochondrial function, reduce inflammation, and improve cognitive outcomes [21]. Replenishing NAD^+^ also exerts anti-inflammatory effects by preventing the activation of tissue-resident immune cells, thereby reducing inflammation [26]. In particular, recent studies have also demonstrated that NR ameliorates glial activation in LPS-induced neuroinflammation and alcohol-induced depression-like behavior mouse models [27, 28]. In addition, in AD mouse models, NR supplementation inhibited disease-associated neuroinflammation, decreased the numbers of activated microglia and astrocytes, and reduced pro-inflammatory cytokines [25, 29].

Despite these promising findings, there remain significant research gaps regarding the direct effects of NR on microglial phenotype and transcriptional regulation during normal aging. Understanding how NR influences microglial transcriptional and functional changes in aged brains is crucial to developing targeted therapies for age-related cognitive decline. This study aims to address this gap by investigating the effects of NR on microglial health, transcriptional profiles, and cognitive function in aged mice. This research has the potential to advance our understanding of NAD^+^ replenishment as a therapeutic strategy for age-related cognitive decline. We identify novel pathways that may support cognitive health and delay neurodegenerative processes in aging populations by elucidating the mechanisms by which NR modulates microglial phenotype and function. These findings may contribute to the development of targeted interventions to preserve cognitive function in aging individuals.

## Methods

### Mice

Animal studies were conducted in compliance with the University at Buffalo Institutional Animal Care and Use Committees (IACUC) guidelines #PROTO201900039. Animal housing and husbandry followed institutional and NIH standards for the care and use of laboratory animals. Every effort was made to minimize animal suffering and to use the minimum number of animals necessary to achieve statistical validity. Male C57BL/6NIA mice aged 4 and 20 months were obtained from the NIA mouse colony maintained at Charles River Laboratories (Wilmington, MA). The mice were kept in a 12-h light and 12-h dark cycle and had access to water and chow at all times. Mice were randomly assigned to Aged and Aged + NR groups based on their body composition, treadmill endurance, and open field activity, the parameters that were significantly improved after NR supplementation in our previous study [23]. For 8 weeks, the 22-month-old mice were given either NR chow (*n* = 15) at 4 g/kg chow, corresponding to 400 mg/kg mouse weight, or placebo chow (*n* = 15). The placebo and NR chow were made by Dyets, Inc. (Pittsburgh, PA). Additionally, ten 6-month-old C57BL/6NIA male mice were used to acquire tissues and microglia for comparison.

### Cognition tests

The assessments were conducted with appropriate rest intervals between tests. All investigators were blinded to supplementation groups, and the same investigator carried out the same experiments at each time point. Additionally, all experiments were performed during daylight hours.

#### Nest building

For the nest building evaluation, 15 g of nesting material consisting of shredded paper and a cotton pad was placed in new cages with individually housed mice. The quality of the nest was assessed after 24 and 48 h, using a 0–5 rating scale according to [30]. A rating of 0 indicated no nest, and a rating of 5 indicated a nest with four walls and covering.

#### Y-maze spontaneous alteration

The mice were allowed to acclimate to their home cages in the testing room for approximately 30 min before the assessment. They were then placed at the center of the Y-maze apparatus, where they explored the A, B, or C arms for 10 min. The recorded 10-min videos were analyzed to determine the total number of alterations.

#### Novel object recognition

For the initial observation phase, mice were placed in a shoebox-style cage (46 × 25 × 20 cm) containing two identical familiar objects (plastic blocks, ~ 4 cm in height and ~ 3 cm in width), and exploration time was recorded over 10 min. After a 3-h retention interval, one of the familiar objects was replaced with a novel object of a different shape and size (~ 4–6 cm), and exploration was recorded for 10 min. Objects were placed in opposite corners of the arena in consistent positions across trials. ANY-Maze software was used for automated tracking and analysis. An investigation zone (10 mm radius around each object) was defined, requiring the mouse to be oriented toward the object; climbing or sitting on objects was excluded from scoring. Total exploration time for familiar and novel objects was extracted for each animal.

#### Animal tissue processing and immunofluorescence analysis

Mice were anesthetized with isoflurane (2–3%) prior to perfusion, followed by transcardial perfusion with PBS and 4% paraformaldehyde (PFA) in PBS. Brains were embedded in OCT and sectioned coronally at 16 µm thickness using a cryostat. The brain sections were washed in PBS and permeabilized for 30 min in 0.3% Triton X-100 and then blocked for 1 h at room temperature in 5% normal donkey serum with 0.3% Triton X-100. The following primary antibodies were used: goat anti-IBA1 (1:100, 011–27991, Fujifilm Wako, Richmond, VA), chicken anti-GFAP (1:200, ab4674, Abcam, Waltham, MA), and rabbit anti-LPL (1:400, NBP2-58,366, Novus, Centennial, CO). Primary antibodies were incubated overnight at 4 °C. After washing, sections were incubated in blocking buffer for 1 h at room temperature. After washing, sections were incubated for 1 h at room temperature with species-specific secondary antibodies diluted 1:500: donkey anti-goat Alexa Fluor 647 (A-21447, Thermo Fisher, Waltham, MA), donkey anti-rabbit Alexa Fluor 555 (A-31572, Thermo Fisher, Waltham, MA), or donkey anti-chicken Alexa Fluor 488 (A-78948, Thermo Fisher, Waltham, MA). Sections were mounted using VECTASHIELD antifade mounting medium without DAPI. Images were acquired on a Keyence BZ-X810 fluorescence microscope (Keyence, Itasca, IL) at 20 × magnification. For each marker, mean fluorescence intensity (MFI) was quantified using ImageJ from at least two brain sections per animal. Quantification was performed in three different regions within the cortex and hippocampus per section. To ensure comparability across groups, we used identical exposure times and applied the same lookup table (LUT) settings for all IF images. This approach minimizes bias and allows direct visual comparison between groups. For the LPL quantification, the areas of interest were drawn around IBA1^+^ cells using automated thresholding in ImageJ/Fiji. The mean fluorescence intensity (MFI) of LPL within these ROIs was then measured. All analyses were conducted by an investigator blinded to the experimental groups.

#### Microglia isolation

Microglia were isolated from whole brain using the Miltenyi adult brain dissociation and CD11b microglia isolation kits (Miltenyi Biotec, Gaithersburg, MD) with the Miltenyi gentleMACS tissue dissociator and autoMACSpro system (Miltenyi Biotec, Gaithersburg, MD), following the manufacturer’s protocol. Antibodies were used to confirm the highly enriched microglial population using a BD LSR II flow cytometer (BD Biosciences, Franklin Lakes, NJ).

#### Bulk RNA sequencing

Total RNA was extracted from microglia using the RNeasy Mini Kit (Qiagen, USA). Whole-genome sequencing was performed on the NovaSeq 6000 System (Illumina Inc., San Diego, CA), conducted at the Genomics and Bioinformatics Core at the New York State Center of Excellence for Bioinformatics and Life Sciences, University at Buffalo, NY. To assess the sequencing quality and identify potential contamination, we employed FastQC and FastQ. Differentially expressed genes were identified using the Bioconductor package DESeq2 version 1.32.0.

#### Gene ontology enrichment analysis

After conducting the differential expression analysis, the gene list was refined and processed by removing blank values and applying a significant p-adj filter (< 0.05). Genes with a log2fold change greater than 0 were classified as upregulated, while those with less than 0 were downregulated. This refined gene list was then used for Gene Ontology (GO) enrichment analysis. The ClusterProfiler package in R software was used to carry out the GO enrichment analysis. First, a list of genes of interest consisting of gene symbols was prepared, and the package was installed and loaded into the R session. Next, the gene list was subjected to GO enrichment analysis using the enrichGO function provided by ClusterProfiler. This function identified GO terms that were significantly enriched among the genes of interest compared to the background gene set. The enrichGO function uses the hypergeometric test and adjusts for multiple testing using the Benjamini–Hochberg method. The resulting enriched GO terms were ranked based on their enrichment significance, determined by the adjusted *p*-value. The top enriched GO terms were then visualized using bubble plots that summarize the enriched terms and their associated gene counts.

#### Metabolic pipeline analysis

The metabolic pipeline [31] utilized the output from the differentially expressed genes (DEGs) analysis. Briefly, a score was calculated for each gene, resulting from the product of the negative logarithm of the adjusted p-value and the log fold change (logFC). Absolute value scores were subsequently derived by taking the absolute value of these calculated scores for each gene. Scores were then constructed for each of 114 pathways, with a bootstrapping approach utilized to assess the statistical significance of the transcriptional dysregulation. This allowed for visualization of the results in multiple DEGs as heatmaps and provided a premise for pathway modeling.

#### Pathway modeling with Cytoscape

Pathway maps were created using Cytoscape software (version 3.8.2), particularly VizMapper functions. These maps were modified based on existing pathways from WikiPathways (version 3.3.7). The DEG analysis output from DESeq2 informed the shading of transcripts in the pathways: red indicates a positive fold change, blue indicates a negative fold change, white represents non-statistically significant values, and gray signifies those not measured. This setup facilitated the visualization of transcripts (represented as triangles), proteins (depicted as ellipses), and metabolites (shown as rounded rectangles) within a single pathway.

#### Data analysis

The statistical analyses were performed using GraphPad Prism 10.4.0 from San Diego, CA. The data are presented as mean ± standard deviation. Normality of data distribution was assessed using the Shapiro–Wilk test before applying ANOVA. Two-way repeated measures analysis of variance (ANOVA) was performed to compare the baseline and endpoint within and between the Young, Aged, and Aged + NR groups. One-way ANOVA was conducted to compare the endpoint assessments between three groups (Young, Aged, and Aged + NR). An unpaired Student’s t-test was performed when comparing the two groups. Statistically significant values were considered when p-values were less than 0.05.

## Results

### NR increases learning and memory in normally aged mice

**Fig. 1:**
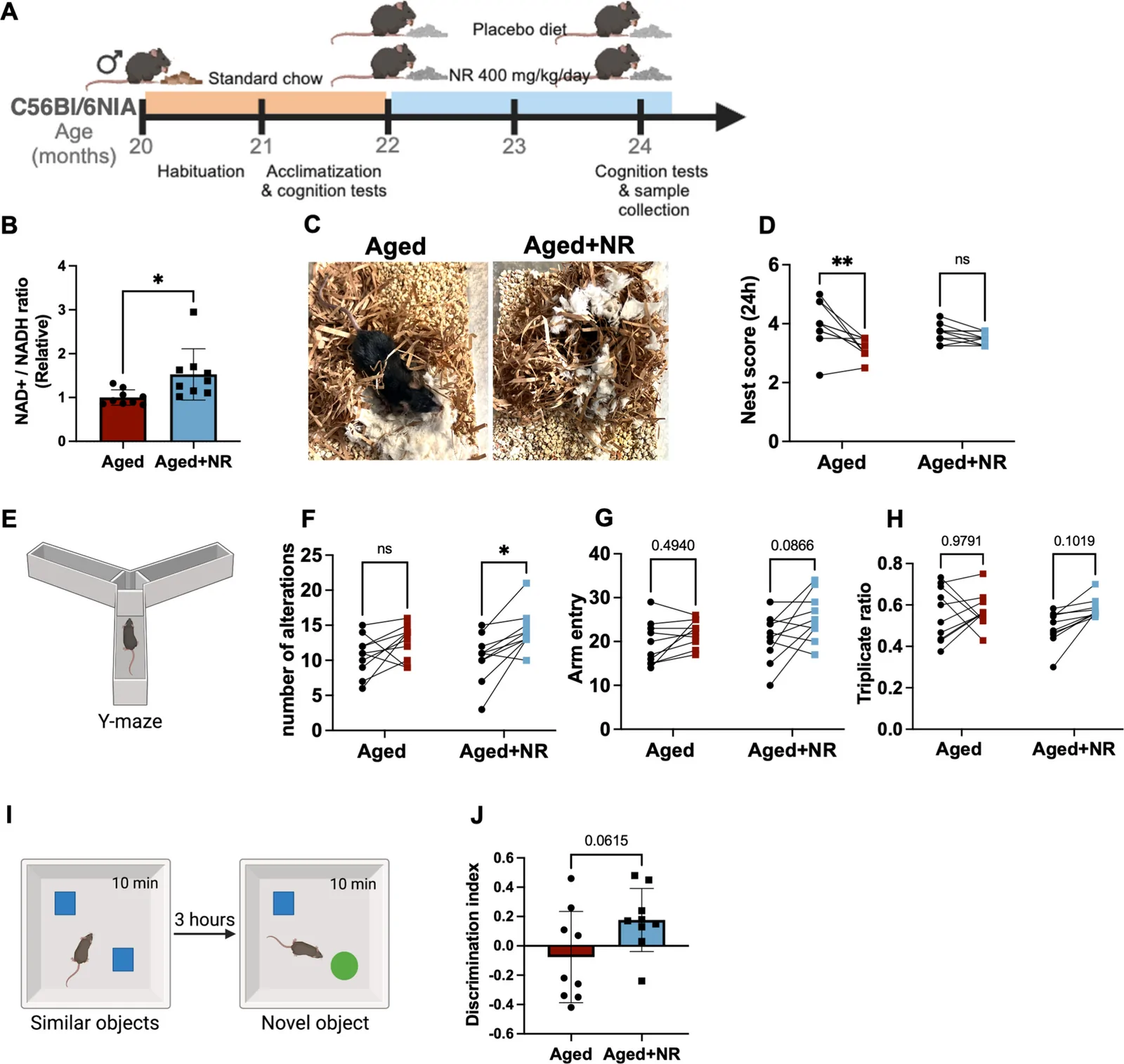
NAD replenishment improved cognition in aged mice. Twenty-two-month-old C57Bl6/NIA male mice were supplemented with NR at a dose of 400 mg/kg/day in chow for 8 weeks. **A** Schematic representation of the study methodology. Eight weeks of NR supplementation increased the NAD^+^/NADH ratio in the brain (**B**). Nesting behavior was assessed with images of nests from Aged control and Aged + NR mice (**C**) and corresponding nest scores (**D**). Spatial working memory was evaluated using the Y-maze platform (**E**), and their spontaneous alterations were analyzed using the number of alterations (**F**), number of arm entries (**G**), and triplicate ratio (**H**). Recognition memory was tested using the novel object recognition test (**I**), and the preferences for the novel object were presented as discrimination index (**J**). Unpaired Student’s *t*-tests were performed to compare the Aged and Aged + NR groups. Statistical analyses were performed using two-way ANOVA with Tukey’s post hoc test for multiple comparisons. Data are presented as mean ± standard deviation with * indicating *p* < 0.05 and ** indicating *p* < 0.001

Our previous study on 15-month-old male mice showed that short-term (4 weeks) NR supplementation increased muscle NAD^+^ levels and muscle fiber quality [23]. To determine whether NAD^+^ depletion contributes to age-related cognitive decline in aged mice, we supplemented chow without or with nicotinamide riboside (NR) at 400 mg/kg/day to 22-month-old mice for 8 weeks. Behavioral analyses were conducted at the baseline (before supplementing mice with NR) and at the endpoint, followed by euthanasia for brain tissue processing and microglial isolation for RNA sequencing (Fig. 1A). The aged mice in the NR group showed a modest (statistically non-significant) change in body weight (Supplementary Fig. 1A), but there were no differences in their percentage of body fat (Supplementary Fig. 1B) and percentage of body lean mass (Supplementary Fig. 1C). However, 8 weeks of NR supplementation significantly increased the NAD^+^/NADH ratio in the cortices of the aged mice (Fig. 1B). In these mice, we found that NR supplementation enhanced cognitive function. Although mice were randomly allocated to the groups, baseline differences were observed, which likely reflect natural heterogeneity in aged mice. Between 22 and 24 months, aged mice exhibited a significant decline in nest-building ability (Fig. 1C), which is comparable to an instrumental activity of daily living, whereas NR supplementation prevented this decline (Fig. 1D). In the Y-maze spontaneous alternation test (Fig. 1E), an indicator of working memory, the Aged + NR group mice made significantly more alternations at 24 months compared to 22 months, whereas the aged mice without NR supplementation did not improve (Fig. 1F). In addition, the number of arm entries and triplicate ratio showed a trend toward improvement in NR-supplemented mice (Fig. 1G and H). Similarly, in the novel object recognition test (Fig. 1I), the mice in the NR group trended to spend more time exploring novel objects, analyzed using the triplicate ratio (Fig. 1J), suggesting potential improvements in recognition memory.

In addition, we performed a battery of physical performance tests, including gait speed, treadmill endurance, and an open field activity monitor. We found that all the mice in the Aged + NR group appeared to exhibit greater treadmill endurance than the control mice (Supplement Fig. 1D and E). However, there was no difference in gait speed between the Aged and Aged + NR groups (Supplement Fig. 1F and G). Interestingly, in the open field activity monitoring (Supplement Fig. 1H), Aged + NR mice exhibited increased rearing behavior (Supplement Fig. 1I), suggesting heightened exploratory activity. There were no significant differences in the total distance traveled in the aged mice, with or without NR (Supplement Fig. 1J).

### NR attenuates age-related neuroinflammation by reducing microglial and astrocyte activation

**Fig. 2:**
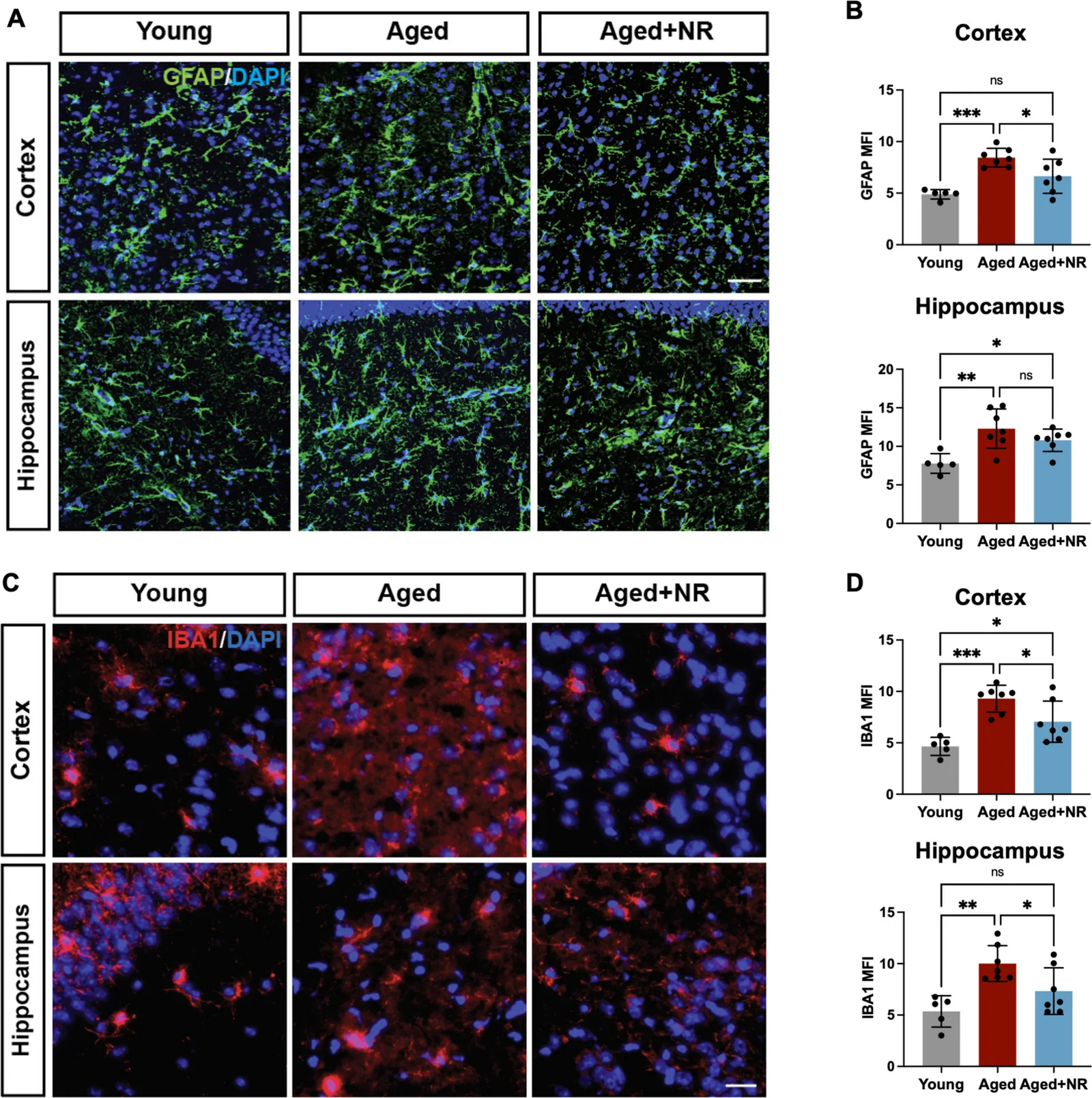
NR reduces astrocyte and microglia activation in aged mice. **A** Representative images of GFAP (green) staining in the cortex and hippocampus of Young (*n* = 5), Aged (*n* = 7), and Aged + NR (*n* = 7) mice. **B** Quantification of GFAP mean fluorescence intensity (MFI) in three regions of the cortex and hippocampus. **C** Representative images of IBA1 (red) staining in the cortex and hippocampus. **D** Quantification of IBA1 MFI in three regions of the cortex and hippocampus. A total of three mice per group were analyzed. Statistical analyses were performed using one-way ANOVA with Tukey’s post hoc test for multiple comparisons. Data are presented as mean ± standard deviation with * indicating *p* < 0.05, ** indicating p<0.001 and *** indicating *p* < 0.0001. Scale, 50 µm (**A**) and 20 µm (**C**)

Aging is associated with increased neuroinflammation, which contributes to cognitive decline. Activated microglia and astrocytes release inflammatory mediators, exacerbating neuroinflammation. To assess glial activation, we analyzed the expression of IBA1 (microglia) and GFAP (astrocytes) in the cortex and hippocampus of Young, Aged, and Aged + NR mice. In aged mice, GFAP expression was significantly elevated in both the cortex and hippocampus compared to young mice, indicating astrocyte activation (Fig. 2A). Quantitative analysis of GFAP mean fluorescence intensity (MFI) confirmed this increase, whereas NR supplementation significantly reduced GFAP expression in the cortex, restoring it toward levels observed in young mice (Fig. 2B). Similarly, aged mice exhibited a marked increase in IBA1 expression in both brain regions, reflecting microglial activation (Fig. 2C). Quantification of IBA1 MFI demonstrated that NR supplementation significantly reduced the age-related increase in IBA1 expression in the cortex and hippocampus, restoring it toward levels observed in young mice (Fig. 2D).

### NR supplementation modulates gene expression in the microglia of aged mice

**Fig. 3:**
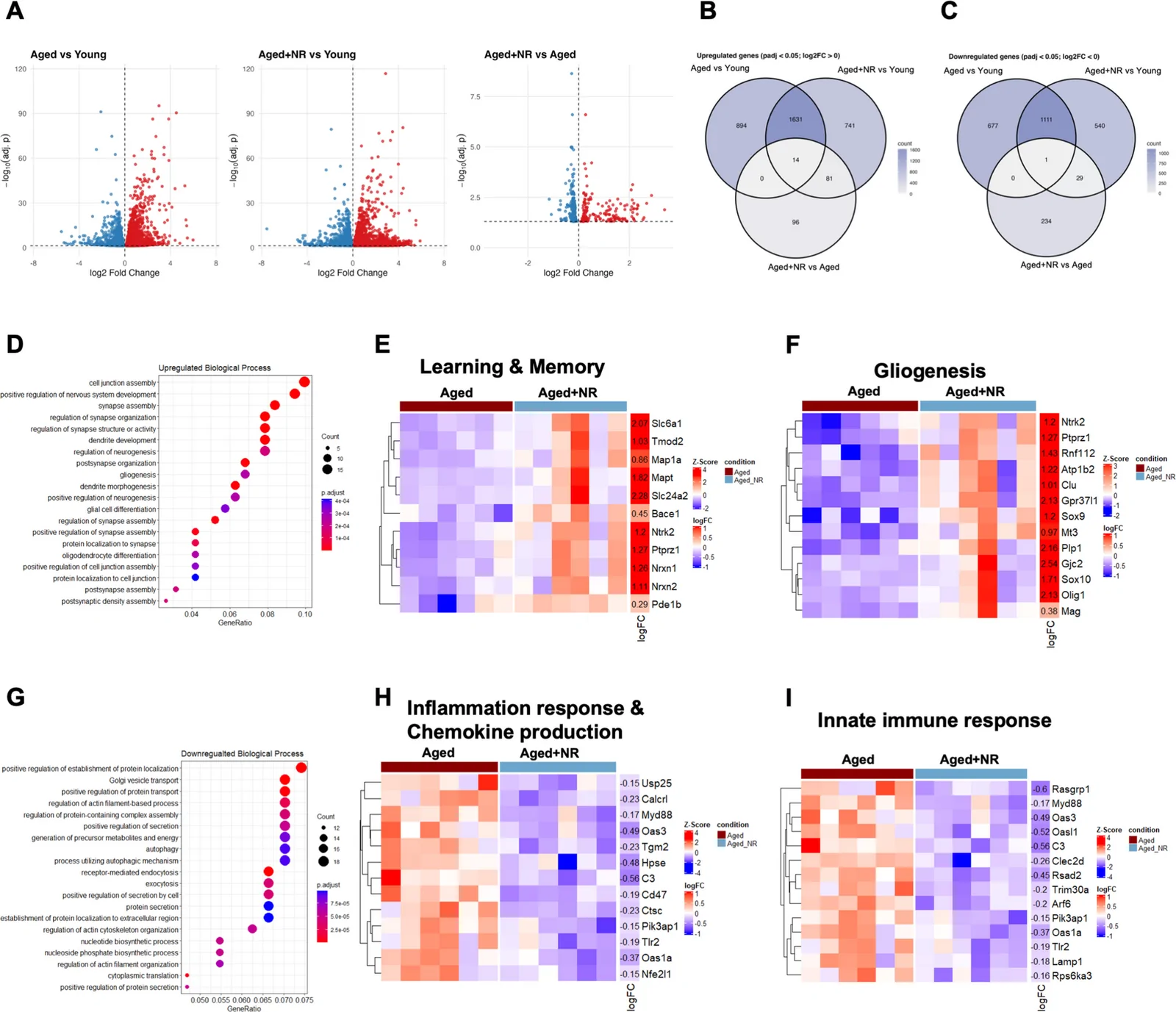
NR upregulates genes related to learning and memory and downregulates neuroinflammation in aged mice. RNA-seq analysis was performed on magnetically sorted microglia from Young, Aged, and Aged + NR mice. **A** Volcano plots show DEGs in Aged vs Young (left), Aged + NR vs Young (middle), and Aged + NR vs Aged (right). Each point represents a single gene, with significantly upregulated genes shown in red and downregulated genes in blue (adjusted *p* < 0.05, log2 fold change threshold = 0). **B** Venn diagrams illustrate the upregulated and downregulated genes across all three comparisons: Aged versus Young, Aged + NR versus Young, and Aged + NR versus Aged. Numbers indicate the counts of genes in each region of the diagram. **C** Heatmap of key differentially expressed genes, showing relative expression across samples. **D** Gene Ontology (GO) analysis of upregulated biological processes, with corresponding heatmaps for genes associated with learning and memory (**E**) and gliogenesis (**F**). **G** GO term analysis of downregulated biological processes, with corresponding heatmaps for genes involved in inflammatory response and chemokine production (**H**) and innate immune response (**I**)

To further dissect the transcriptional alterations across groups, we performed pairwise differential expression analyses of microglia from Young, Aged, and Aged + NR mice and displayed them in a side-by-side volcano plot (A). Aging induced extensive transcriptional changes, with 4,328 differentially expressed genes (DEGs) in aged microglia compared to young controls (2,539 upregulated, 1,789 downregulated; adjusted p < 0.05). NR supplementation attenuated a portion of these changes, as reflected by the Aged + NR vs Young comparison, which identified 4,149 DEGs (2,467 upregulated, 1,682 downregulated). Importantly, the direct comparison between Aged + NR and Aged microglia revealed 455 DEGs, of which 191 were upregulated and 264 were downregulated in the NR-supplemented group. To further dissect the transcriptional changes in microglia, we compared the overlap of significantly upregulated and downregulated genes across the three experimental groups (Aged vs Young, Aged + NR vs Young, and Aged + NR vs Aged) using a Venn diagram (Fig. 3B and C). For upregulated genes, we identified 894 unique to Aged vs Young, 741 unique to Aged + NR vs Young, and 96 unique to Aged + NR vs Aged. Notably, 1,631 genes were commonly upregulated in both Aged vs Young and Aged + NR vs Young, with 14 genes upregulated across all three comparisons. For downregulated genes, 677 were unique to Aged vs Young, 540 to Aged + NR vs Young, and 234 to Aged + NR vs Aged. A total of 1,111 downregulated genes were shared between Aged vs Young and Aged + NR vs Young, whereas only a single gene (*Ctsc*) was consistently downregulated across all three comparisons. To further assess global transcriptional shifts, PCA analysis (Supplementary Fig. 3D) demonstrated clear separation of young, aged, and Aged + NR groups, with NR supplementation shifting aged microglia toward an intermediate state between aged and young profiles. In addition, hierarchical clustering (Supplementary Fig. 3C) revealed that aged microglia exhibit widespread transcriptional alterations compared to young controls, many of which were partially restored by NR supplementation. Next, we performed gene ontology (GO) analysis of the differentially expressed genes, indicating that NR upregulated biological processes (Fig. 3D) related to learning and memory (Fig. 3E), and to gliogenesis (Fig. 3F). Conversely, genes related to neuroinflammation and innate immunity were downregulated in microglia from NR-supplemented aged mice compared to controls (Fig. 3G-I). To validate the RNA-seq results, we performed RT-qPCR on microglial mRNA. Consistent with the RNA-seq findings, aged microglia showed increased expression of *Lpl, Apoe, Trem2, C1qb, Tlr7,* and *Sparcl1* compared to young controls (Supplementary Fig. 4A). These results confirm the transcriptomic alterations identified by bulk RNA sequencing.

### NR preserves homeostatic microglial gene expression and reduces age-dependent and disease-associated phenotypes

**Fig. 4:**
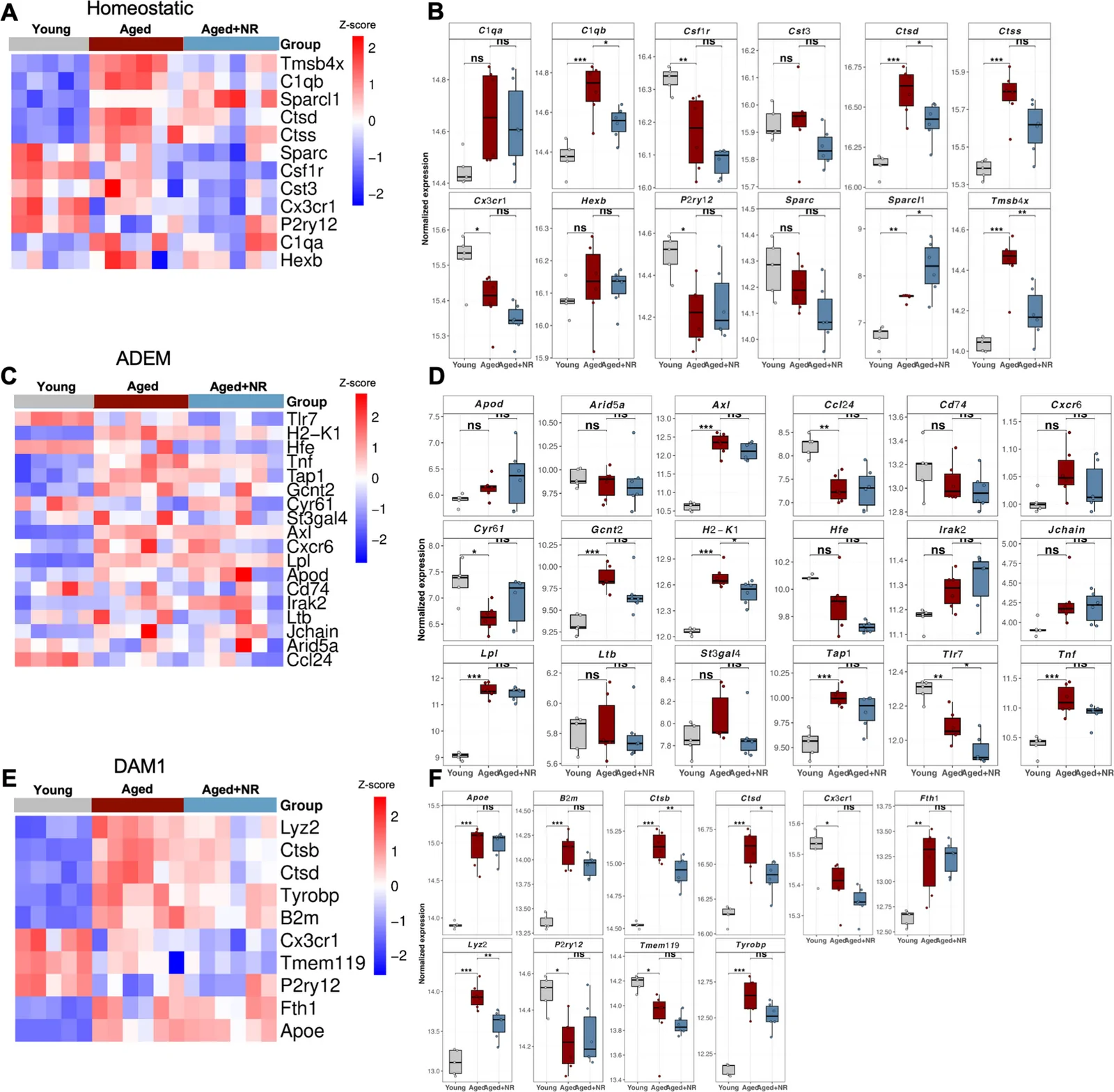
NR modulates microglial gene expression in homeostatic, age-dependent and disease-associated phenotypes in aged mice. **A** Heatmap comparing gene expression profiles of homeostatic microglia in Aged and Aged + NR groups. **B** Box plots of the expression levels of homeostatic microglial genes in Aged and Aged + NR groups. **C** Heatmap comparing gene expression profiles of the age-dependent microglia (ADEM) phenotype in Aged and Aged + NR groups. **D** Box plots for the top 5 genes based on *p*-value in ADEM. **E** Heatmap comparing gene expression profiles of disease-associated microglia 1 (DAM1) in Aged and Aged + NR groups. **F** Box plots for the top 5 genes based on *p*-value in the DAM1 phenotype. Statistical comparisons were performed using one-way ANOVA with Tukey’s post hoc test. Data are presented as mean ± standard deviation, with * indicating *p* < 0.05, ** indicating p<0.001 and *** indicating *p* < 0.0001

Microglia play a crucial role in maintaining brain homeostasis; however, aging and neurodegenerative diseases transition them to pro-inflammatory states, including age-dependent microglia (ADEM) and disease-associated microglia (DAM) [31]. DAM is further divided into two stages: DAM1, which represents an early activation state, and DAM2, characterized by a more pronounced inflammatory profile. These genes highlight pathways most strongly associated with aging, neuroinflammation, and microglial activation, and thus provide a focused view of NR’s impact on age-driven and disease-associated transcriptional changes in microglia. By emphasizing genes that are established markers of homeostatic, ADEM, DAM1, and DAM2 states, we aimed to provide a representative overview of well-characterized pathways most relevant to microglial aging and NAD^+^-based interventions. Aging markedly altered microglial gene expression compared to young controls. For homeostatic genes [32], aged mice showed increased expression of *C1qa, C1qb, Ctss,* and *Ctsd*, along with reduced Sparcl1 and Tmsb4x relative to young mice (Fig. 4A). NR supplementation significantly lowered *C1qa, C1qb, Ctss,* and *Ctsd* expression while restoring *Sparcl1* and *Tmsb4x* levels, shifting them toward the young phenotype (Fig. 4B). Next, ADEM-associated genes, defined previously [33], were also elevated in aged microglia, with *Apod, Arid5a, Axl, Cd14, Cd74, Cxcr6, Cyb1, Gcnt2, H2-k1, Hfe, Irak2, Jchain, Lpl, Ltb, St3gal4, Tap1, Tlr7,* and *Tnf* significantly upregulated relative to young mice (Fig. 4C). NR treatment reduced the expression of *Tlr7* and *H2-k1*, bringing levels closer to those observed in young microglia (Fig. 4D). For DAM1-associated genes, defined previously [32], aged mice displayed significantly increased expression of *Apoe, B2m, Ctsb, Ctsd, Fth1, Lyz2,* and *Tyrobp* with decreased expression of Cx3cr1, P2ry12, and Tmem119 compared to young controls. This age-induced increase in *Lyz2, Ctsb,* and *Ctsd* was significantly reduced by NR supplementation, again shifting expression toward the young phenotype (Fig. 4F). Aging also significantly increased the expression of DAM2 markers such as *Axl, Ccl6, Cd9, Clec7a, Csf1, Cst7, Ctsl, Itgax, Lilrb4, Lpl, Timp2,* and *Trem2* (Supplementary Fig. 3A). NR supplementation significantly reduced the expression of *Timp2*, and the remaining genes shifted toward the microglial gene expression profile of young mice (Supplementary Fig. 3B). To assess whether aging or NR supplementation altered microglial proliferation, we measured *Ki67* transcript levels by RT-qPCR in isolated microglia. Ki67 expression was significantly reduced in aged microglia compared to young controls and was not restored by NR treatment (Supplementary Fig. 4B). These findings indicate that the age-associated decline in microglial proliferation persists despite NR supplementation.

### NR reduces glucose and lipid metabolism in the microglia of aged mice, resembling young mice microglia

**Fig. 5:**
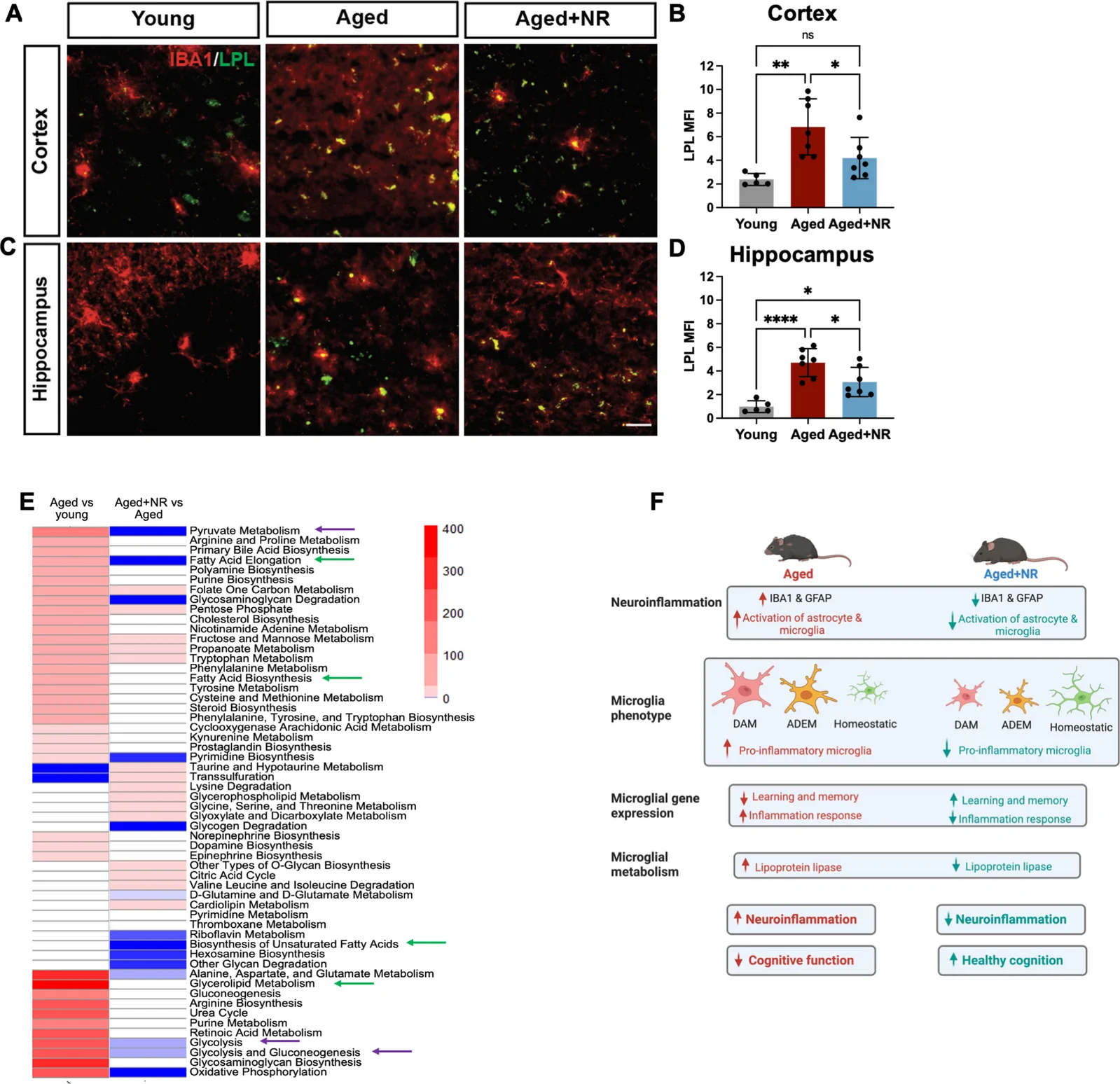
NR reduces lipid metabolism in microglia of aged mice, aligning their metabolic profile with that of young microglia. Representative images of LPL (green), IBA1 (red), and DAPI (blue) immunofluorescence staining of the cortex from Young (*n* = 5), Aged (*n* = 7), and Aged + NR (*n* = 7) mice (**A**). The mean fluorescence intensity (MFI) of LPL in microglia was analyzed in three regions of the cortex (**B**) across three different mice from each group. Representative images of LPL (green), IBA1 (red), and DAPI (blue) immunofluorescence staining of the cortex from the Young, Aged, and Aged + NR groups (**C**). The mean fluorescence intensity (MFI) of LPL in microglia was analyzed in three regions of the hippocampus (**D**) across three different mice from each group. **E** Pathway analysis of differentially expressed genes (DEGs) indicates reductions in glucose metabolism (purple arrows) and fatty acid metabolism (green arrows) in Aged + NR microglia compared to aged microglia. **F** A summary schematic illustrating the overall metabolic shift induced by NR supplementation in aged microglia was created using BioRender. Statistical analyses were performed using one-way ANOVA with Tukey’s post hoc test for multiple comparisons. Data are presented as mean ± standard deviation, with * indicating *p* < 0.05, ** indicating p<0.001, and **** indicating *p* < 0.0001. Scale, 20 µm (**A**, **B**)

In homeostatic microglia, glucose metabolism and oxidative phosphorylation (OXPHOS) predominantly support cellular maintenance and surveillance [34]. DAM phenotypes have a metabolic shift toward increased lipid accumulation and fatty acid metabolism, fueling pro-inflammatory activities [35]. Immunofluorescence staining for lipoprotein lipase (LPL) in the cortex of aged mice revealed elevated LPL expression (Fig. 5A and B), consistent with this metabolic change. In contrast, NR supplementation significantly reduced LPL levels in the cortex of aged mice, aligning them with the lower expression seen in young mice (Fig. 5A and B). Similarly, the hippocampus of aged mice exhibited higher LPL expression in microglia (Fig. 5C and D), which was also reduced with NR supplementation, restoring levels to those comparable to young mice. In the aging or AD brain, microglia exhibit increased glucose and lipid metabolism, contributing to a pro-inflammatory state associated with neuroinflammation [35–39]. Further, metabolic pipeline analysis of differentially expressed genes (DEGs) in microglia revealed that the glucose and lipid metabolism and oxidative phosphorylation were elevated in aged mice compared to young mice (Fig. 5E). Surprisingly, NR supplementation downregulated the glucose and fatty acid metabolism in aged mice (Fig. 5E). The schematic in Fig. 5F illustrates the impact of NR supplementation on the shift in microglial phenotype, metabolic reprogramming, and neuroinflammation, with concomitant changes in cognitive function.

## Discussion

Findings from this study reveal the beneficial impacts of NR supplementation on cognition, neuroinflammation, microglial phenotype, and microglial metabolism in aged wild-type mice. In particular, aged mice displayed a marked impairment in cognitive function assessed using a battery of tests, increased neuroinflammation (microglia and astrocyte activation markers, IBA1 and GFAP, respectively), a shift in microglia gene expression toward the DAM phenotype, and a transition in microglial fatty acid and glucose metabolism toward a phenotype consistent with neurodegenerative disease. However, NR supplementation mitigated these cognitive deficits and modulated microglial gene expression and metabolism to resemble microglia from younger mice, thereby showcasing its potential to rejuvenate age-related cognitive decline and microglial dysfunction. These findings lay a foundation for additional pre-clinical and clinical research in this area using NR or NAD^+^ precursors for treating or preventing age-related cognitive decline and dementia.

Increases in chronic inflammation and oxidative stress deregulate NAD^+^ metabolism, decreasing NAD^+^ during aging [20]. Given that NAD^+^ plays a central role in cellular metabolism and energy homeostasis, we also examined whether NR supplementation affected body composition in our aged mice. Eight weeks of NR in aged mice resulted in a modest, though not statistically significant, reduction in body weight compared to placebo-treated aged mice (Supplementary Fig. 1A—C). This finding extends our previous work demonstrating that NR supplementation (300–600 mg/kg/day for 4 weeks) significantly decreased body weight in 15-month-old middle-aged mice [23]. However, in contrast to the significant effects observed in middle-aged mice, the lack of statistical significance in body weight changes in our 24-month-old mice likely reflects the advanced age of the animals, where metabolic responsiveness to interventions may be diminished. To our knowledge, no other studies have specifically examined the effects of NR supplementation on body composition in normally aged wild-type mice of this advanced age. Long-term NMN supplementation (a related NAD + precursor) suppressed age-associated body weight gain in younger mice over 12 months [40], while NR supplementation significantly reduced body weight gain in younger mice fed a high-fat diet [41, 42]. In addition, we did not observe any significant difference in food consumption (data not shown) between NR and placebo-supplemented aged mice. Although changes in body composition were not captured in our aged mice, we focused our investigation on brain tissue, where NAD + depletion has particularly pronounced effects due to the exceptionally high energy demands of neural tissue. Because, even in middle age, the hippocampus in mice has approximately 40% lower NAD^+^ expression than in younger ages [43]. NR supplementation increased cerebral NAD^+^ in middle-aged adults [44] and also elevated the NAD^+^/NADH ratio in the brain cortex of normally aged 18-month-old mice supplemented for 3 months [19]. In addition, supplementation of AD mice with NR increased the NAD^+^/NADH ratio in the cortex [19]. We now show for the first time that NR supplementation increases the NAD^+^/NADH ratio in the cortices of 24-month-old mice. Replenishing NAD^+^ levels using NR enhances memory in 14-month-old middle-aged wild-type mice [45] and in AD mouse models [19, 45, 46]. In contrast, 3 months of NR supplementation did not improve cognition in 16 to 18-month-old wild-type mice [19]. Here, we found that supplementation with NR for 8 weeks in 22-month-old mice prevented age-related decline in nest-building ability (a surrogate marker for instrumental activities of daily living [47] and increased Y-maze spontaneous alternations (indicating improved spatial working memory). Whereas a strong trend toward an increase in novel object recognition (indicating enhanced spatial recognition memory) was observed after NR supplementation in aged mice. Previous studies in aged WT and AD mice have reported variable NOR results depending on model and task parameters. In aged WT mice, working memory remains intact up to 28 months, while long-term memory (24 h delay) shows impairment only at advanced age [48], with additional variability from strain differences and exploration dynamics [49]. Our study used a 3-h delay, whereas Fahlstrom et al. demonstrated clear deficits using 24-h delays, suggesting longer intervals may be more sensitive for detecting age-related impairments. AD models show more consistent deficits across conditions, and NAD^+^ precursors (NR/NMN) reliably improve cognitive performance, including object recognition in AD models [25, 50]. We suggest that future studies consider incorporating both short-term (~ 3-h) and long-term (24-h) delay intervals to comprehensively assess the effects of NR supplementation on recognition memory in aged mice, where the cognitive deficit is minimal. A recent meta-analysis has shown that physical activity is associated with improved late-life cognition [51]. We and others have demonstrated that NR increases physical performance in middle-aged and aged mice [23, 24]. In the aged mice, NR supplementation increased treadmill endurance and exploratory behavior, but no difference in gait speed.

Multiple factors influence age-related cognitive decline, prominently involving neuroinflammation, which involves high levels of pro-inflammatory cytokines and activation of glial cells like microglia and astrocytes [52, 53]. This is especially evident in the elevated expression of markers for activated microglia and astrocytes in cortex and hippocampus regions in AD mouse models [19, 45]. We observed increased expression of IBA1 and GFAP in 24-month-old wild-type mice compared to the 6-month-old young mice in the cortex and hippocampus, consistent with age-related activation of microglia and astrocytes. However, NR supplementation in the aged mice reduced the expression of IBA1 and GFAP in these regions, restoring glial inflammation or neuroinflammation to levels observed in young mice. This is consistent with a previous study in AD mouse models, which showed that NR decreased the GFAP and IBA1 mean fluorescent intensities of activated astrocytes and microglia, respectively [25]. Collectively, our data extend these observations to normal aging and suggest that NR supplementation in aged mice restores NAD^+^/NADH ratios and reduces chronic glial activation, thereby potentially lowering the inflammatory burden that contributes to cognitive decline.

Microglia are recognized as the first cells in the brain to respond to both neuroinflammation and systemic inflammation [54]. Therefore, it is important to understand the cellular/molecular changes in microglia during aging and the impact of NAD^+^ replenishment on aged microglia. We performed bulk RNA sequencing of microglia from the brains of young, aged, and Aged + NR groups and found increased expression of genes related to neuroinflammation, microglial activation, and cognitive decline in aged compared to young mice (Supplementary Fig. 2A, B, and C). In contrast, NR supplementation significantly downregulated genes associated with inflammatory responses, chemokine production, and microglial activation. Similarly, Hou et al. demonstrated that NR suppressed neuroinflammation in AD mice [25]. Interestingly, we also found reduced expression of cognition-related genes observed in aged mice (Supplementary Fig. 2D and E). In contrast, NR supplementation increased the expression of genes associated with learning and memory and with gliogenesis. These results demonstrate that NR supplementation in aged mice leads to significant alterations in microglial gene expression, characterized by the upregulation of genes involved in cognitive function and the downregulation of genes associated with inflammation. This suggests that NR supplementation reshapes the microglial transcriptome, mitigating age-driven transcriptional dysregulation and partially restoring a more youthful gene expression profile. These findings are further supported by PCA and hierarchical clustering (Supplementary Fig. 3C and D), which show that NR supplementation shifts aged microglia toward a younger-like global transcriptomic profile. However, the precise molecular mechanisms linking NAD + replenishment to these transcriptional changes require further investigation. While NR generally restored several homeostatic microglial transcripts toward youthful levels, this subset of genes (*Adgrb1, Ankrd16, Clu, Edil3, Lsamp, Map1a, Nrxn1, Ntrk2, Pabpn1, Peg3, Sparcl1, Tmem47, Tmod2,* and *Ttyh1*) appears to be uniquely induced by NR supplementation in the aged brain. These genes were consistently upregulated in aged microglia compared to young controls and further elevated in Aged + NR mice. Many of these genes regulate synaptic support, stress responses, and cytoskeletal remodeling, suggesting that NR not only counteracts age-driven inflammatory signatures but also induces transcriptional programs potentially related to neuronal support and adaptive remodeling. This indicates that NR supplementation alters/improves the microglial transcriptomic profile in aged mice beyond simply restoring a youthful phenotype.

In continuation of the gene expression changes in microglia, we next explored how NR supplementation affects the functional states of microglia, as defined by their gene expression profiles, and its potential to shift microglia from detrimental states associated with aging and neurodegeneration back to a more homeostatic state. The age-related alterations in microglial gene expression correspond with functional states in response to chronic inflammation and neurodegeneration of AD [2, 55–58]. Functional states of microglia can be defined based on single-cell RNA sequencing and bulk RNA sequencing of microglia from 3-month to 24-month-old mice [2, 55, 56] or from AD mouse models [32, 57] as homeostatic microglia, age-dependent microglia (ADEM), disease-associated microglia (DAM1 and DAM2), and more subsets [59]. By emphasizing genes that are established markers of homeostatic, ADEM, DAM1, and DAM2 states, we aimed to provide a representative overview of well-characterized pathways most relevant to microglial aging and NAD^+^-replenishment based intervention. Aging and AD drive a shift in microglial states from homeostatic to ADEM or DAM characterized by heightened inflammatory responses [32, 57]. In our study, we defined homeostatic, DAM1, and DAM2 microglia phenotypes based on the definition by Keren-Shaul H et al. [32] and ADEM by Li X et al. [33]. NR supplementation reduced the expression of homeostatic genes associated with heightened complement activity (*C1qb*), lysosomal stress (*Ctss* and *Ctsd*), and cytoskeletal gene (*Tmsb4x*), which were upregulated in aged mice compared to young mice. In contrast, NR increased the expression of *Sparcl1*, a neuroprotective gene usually downregulated in aging and neurodegeneration, further supporting that NR helps restore homeostatic microglial function. Importantly, even in the normally aged 24-month-old mice, the ADEM, DAM1, and DAM2 genes are upregulated compared to young mice. Notably, NR supplementation significantly downregulated ADEM, DAM1, and DAM2 phenotype genes involved in inflammatory signaling (*Tlr7*), antigen presentation (*H2-k1*), phagocytic response (*Ctsb*), and lysosomal stress (*Lyz2* and *Timp2*)—all hallmarks of the ADEM and DAM phenotypes. This suggests that NR supplementation helps restore microglial function by suppressing the inflammatory-driven microglial phenotypes associated with aging and neurodegeneration, offering a promising strategy for maintaining microglial health and function during aging and neurodegeneration. Together, these data show that aging drives microglial transcriptional reprogramming across homeostatic, ADEM, and DAM1 states, and that NR supplementation mitigates these changes, partially restoring microglial gene expression toward a more youthful profile (Supplementary Fig. 3C).

Since changes in microglial state are linked to their metabolic demands, we extended our analysis to determine how aging and NR supplementation alter microglial metabolism. Under homeostatic conditions, microglia utilize glycolysis and OXPHOS to meet the required energy demands for their function [34]. In contrast, aging and neurodegenerative conditions induce lipid droplet accumulation, altered lipid metabolism, and elevated glucose utilization in microglia [35–39, 60]. The metabolic pathway analysis of microglial genes in young and 24-month-old wild-type mice revealed a marked age-related shift in overall microglial metabolism toward increased glucose metabolism and lipid metabolism in response to heightened neuroinflammation. Marschallinger et al. demonstrated elevated lipid accumulation in microglia, termed lipid-droplet accumulating microglia, which exhibited phenotypic characteristics of reduced phagocytosis and increased secretion of pro-inflammatory cytokines in 20-month-old wild-type mice, as well as in mouse models of acute and chronic inflammation [35]. Furthermore, previous studies have also shown altered lipid metabolism in the microglia of AD mouse models [32, 61]. Notably, metabolic pathway analysis revealed that the NR supplementation reduced the age-related increase in glycolysis and lipid metabolism in microglia from aged mice. These metabolic pathways are typically upregulated in DAM phenotypes, contributing to their pro-inflammatory state. The observed shift in metabolism indicates that NR transitions microglial metabolism toward a state resembling that of younger or healthier, homeostatic microglia, thereby reducing inflammatory activity. In support, the immunofluorescence staining for LPL and IBA1 in the cortex and hippocampus of aged mice revealed elevated LPL expression, indicative of increased lipid metabolism in aged microglia. In contrast, NR supplementation reduced microglial LPL levels in both regions, restoring them to similar levels observed in younger mice. Our findings indicate that NR supplementation reduces these metabolic shifts and states of microglial activation, bringing microglial energy metabolism closer to that observed in younger, homeostatic microglia. Overall, NR enhances microglial health and helps alleviate neuroinflammatory processes that accelerate cognitive decline related to aging.

While our study provides compelling evidence for the beneficial effects of NR supplementation on aged microglial function and cognition, it is important to acknowledge that we have primarily identified correlative relationships rather than establishing direct causal mechanisms. The observed changes in microglial gene expression and metabolism following NR treatment suggest potential pathways through which NAD^+^ replenishment may exert its effects, but direct mechanistic validation through targeted interventions (such as specific pathway inhibition or genetic manipulation) was not performed in this study. Potential mediators such as Sirtuins, AMPK, or downstream transcriptional regulators may link enhanced NAD^+^ metabolism to shifts in lipid handling and inflammatory pathways, but targeted experiments will be required to test these mechanisms directly. Our future research will focus on dissecting the specific molecular pathways linking NAD^+^ replenishment to microglial reprogramming and determining whether the observed microglial changes are necessary and sufficient for the cognitive benefits observed.

Overall, NR enhances microglial health and helps alleviate neuroinflammatory processes that accelerate cognitive decline related to aging. A key limitation is the exclusive use of male mice; future studies including both sexes are essential to determine sex-specific effects of NR on cognitive aging and microglial phenotypes. Both sexes will be studied because aging and neuroinflammation follow sex-specific trajectories. Aged female mice display more microglial activation, neuroinflammatory gene expression, and disease-associated microglia than males [37, 62]. Conversely, males often experience earlier and more robust systemic inflammatory priming with cognitive deficits [63, 64]. Including both sexes ensures broad applicability and allows us to determine if NAD replenishment offers differential benefits in aged mice. Another limitation is that we did not perform detailed morphometric analyses of microglial morphology (e.g., process length, branching complexity), which are commonly used to characterize activation states. Instead, we relied on IBA1 expression, Ki67, and transcriptomic profiling as indicators of microglial activation, and future studies incorporating morphometric analysis will be valuable to strengthen these findings. In addition, while NR supplementation clearly reshaped microglial gene expression and metabolism, the precise mechanistic links to cognitive outcomes remain to be clarified. Furthermore, young controls were not included in the behavioral assays (Fig. 1), which limits direct validation of the positive cognitive effects of NR supplementation. Although young mice were included in the immunofluorescence and transcriptomic analyses (Figs. 2, 4, and 5), future work will incorporate young controls consistently across behavioral testing to strengthen the interpretation of age-related and NR-mediated effects. Finally, behavioral testing was limited to a core battery, and incorporating additional assays, such as the Morris water maze or radial arm maze, in future studies will help validate and expand upon the cognitive findings.

## Conclusion

NR supplementation in aged wild-type mice prevents declines in nest building and enhances working and recognition memory, which are relevant to instrumental activities of daily living. NR concomitantly improves the microglia gene expression profile, consistent with reduced glial inflammation, increased gliogenesis, and improved memory. Furthermore, NR diminishes age-dependent and disease-associated microglial phenotypes while enhancing the homeostatic state. Additionally, NR suppresses glucose and lipid metabolism pathways, which are typically enhanced with age and AD, thereby resembling microglial metabolism in younger mice. Overall, the study outcomes support NR supplementation as a potent intervention for promoting healthy brain aging. These findings support the translation of this work into clinical settings to ascertain the benefits of NR supplementation for maintaining and enhancing functional and cognitive function during aging.

## Supplementary Information

Below is the link to the electronic supplementary material.

ESM 1 (PDF 818 KB)

## Data Availability

The data supporting the findings of this study can be found either in the main text or can be obtained from the authors upon reasonable request.
