# Supplementary material for: Nicotinamide riboside supplementation restores microglial health and improves cognition in aged male mice
Source: GeroScience. 2025 Nov 22;48(5):6629–46. doi: 10.1007/s11357-025-01959-1 (PMC13601472; doi:10.1007/s11357-025-01959-1)
Supplement: Supplementary file 1 [file 11357_2025_1959_MOESM1_ESM.pdf]

Nicotinamide riboside supplementation restores microglial health and improves cognition in aged male mice

Ramkumar Thiyagarajan[1], Rupadevi Muthaiah[1], Bhavana Sreevelu[1], Owen Treanor[1], Yonas Redae[1], Reem Berman[1], Anna Davis[1], Nanda Yellapu[2], Spencer Rosario[3], Lee D. Chaves[1], Kenneth L. Seldeen[1], and Bruce R. Troen[1*]

[1] Division of Geriatrics, Department of Internal Medicine, Landon Center on Aging, University of Kansas Medical Center, Kansas City, KS, Research Service, Veterans Affairs Kansas City Healthcare System, Kansas City, MO.

[2] ICMR-vector control research center, Indira Nagar Medical Complex, Puducherry, India.

[3] Department of Biostatistics and Bioinformatics and Experimental Therapeutics, Roswell Park Comprehensive Cancer Center, Buffalo, NY.

Supplementary methods:

Body composition: Quantitative magnetic resonance (qMR) was utilized to measure the percentage of total body fat and lean mass on a Bruker LF65 instrument.

Grip strength: Grip strength in mice was measured using a grip force meter (Columbus Instruments, Columbus, OH, USA). The mouse was held by the tail and positioned so that all four limbs made contact with the force meter. Subsequently, the tail was pulled until the mouse lost its grip. Between trials, mice were allowed a 10-second rest period. The maximal force generated in the best three out of five trials was utilized for further analysis.

Gait speed: Gait speed was measured using a specialized setup comprising a 1-meter-long hallway 8 cm wide and 12 cm high leading into a darkened "safe house" chamber. The evaluation was based on the average of 4 repeated trials.

Open field activity: To track the activity of mice in the open field, we utilized transparent 8 square boxes/arenas measuring 40 cm x 40 cm x 35 cm, which were equipped with infrared photobeam arrays from MedAssociates Inc., located in Fairfax, VT, USA. Each mouse was placed within the open field arena and recorded for 35 minutes, during which we calculated the total distance covered in the first five and last 30 minutes, crossings between the center and periphery or between four quadrants, and the number of rearing events (vertical activity) using the software provided by the manufacturer.

RT-qPCR: RNA was isolated from magnetic-sorted microglia using Miltenyi CD11b antibody and AutoMACSpro. cDNA synthesis was performed, and quantitative PCR was carried out using SYBR Green master mix, with gene-specific primers (Supplementary Table 1). Relative expression was normalized to housekeeping genes using the ΔΔCt method.

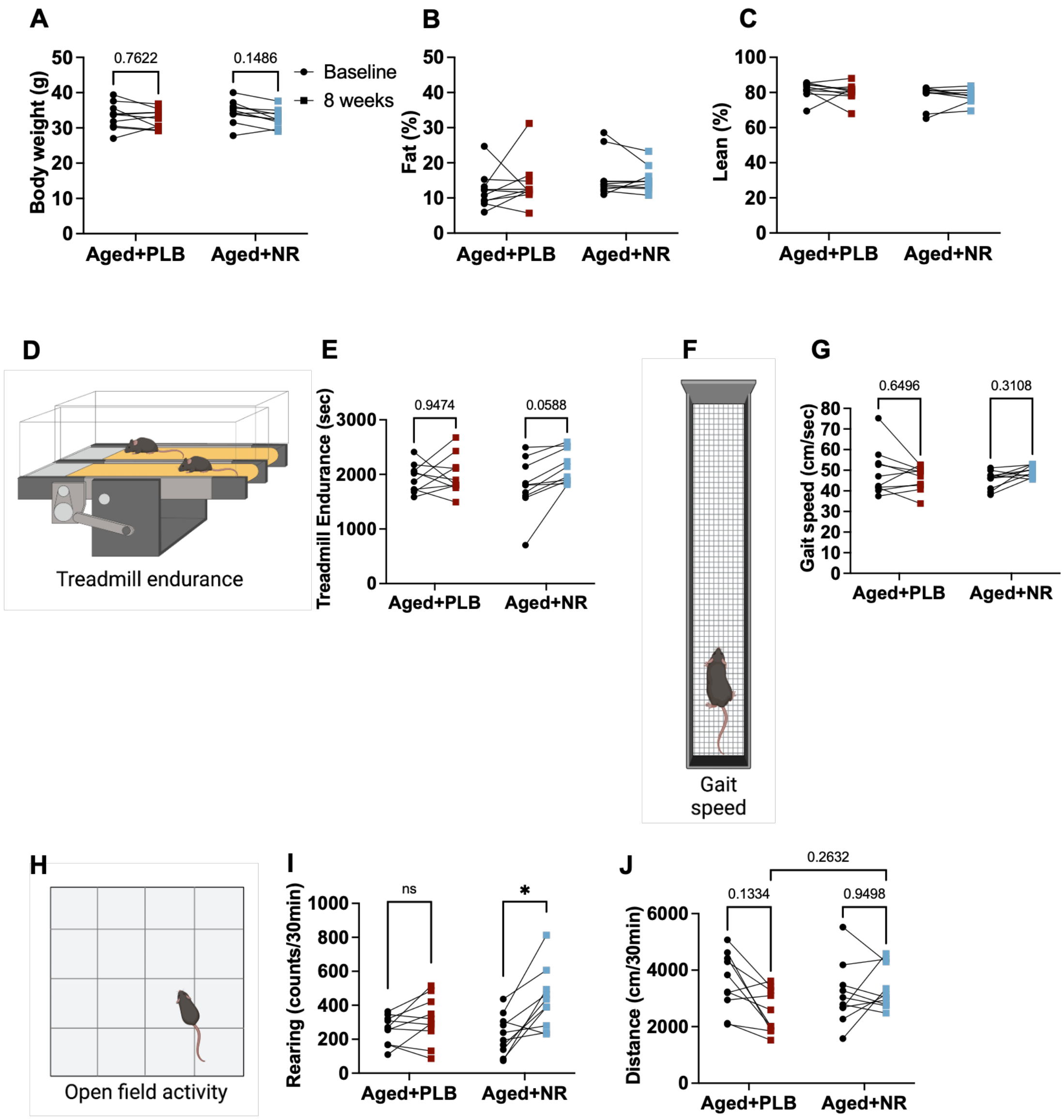

Supplementary Fig. 1: Effects of NR supplementation on body composition and physical performance.

(A) The body weight of the experimental groups showed no significant difference. (B, C) Body fat percentage across groups. (D) Schematic of the treadmill endurance test model. (E) Treadmill endurance, showing a marked increase in aged+NR mice. (F) Schematic representation of gait speed assessment. (G) Gait speed was measured as distance traveled (cm/sec). (H) Open field activity monitoring setup. (I) Rearing behavior and (J) total distance traveled during a 30-minute exploration of the open field arena. A two-way ANOVA was conducted to compare the baseline and endpoint data within and between the groups. Data are presented as mean ± standard deviation with * indicating $p < 0.05$.

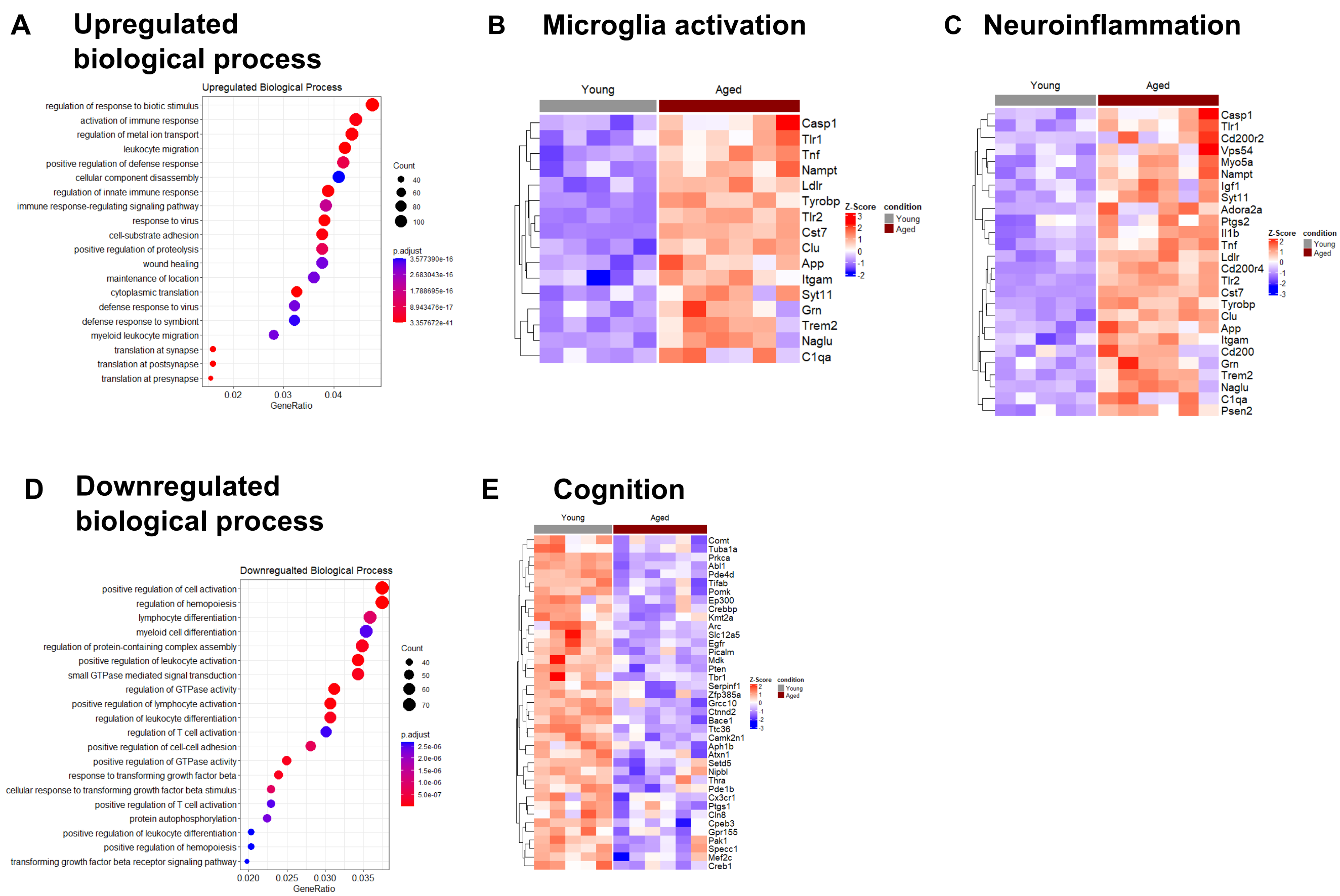

Supplementary Fig. 2: Gene ontology (GO) analysis of differentially expressed genes in aged microglia.

(A) Bubble plot of upregulated biological processes in aged microglia identified by GO term enrichment analysis. Heatmap showing increased expression of genes associated with microglial activation (B) and neuroinflammation (C) GO terms in aged mice. (D) Bubble plot of downregulated biological processes in aged microglia. (E) Heatmap showing reduced expression of genes associated with cognition-related GO terms in aged mice.

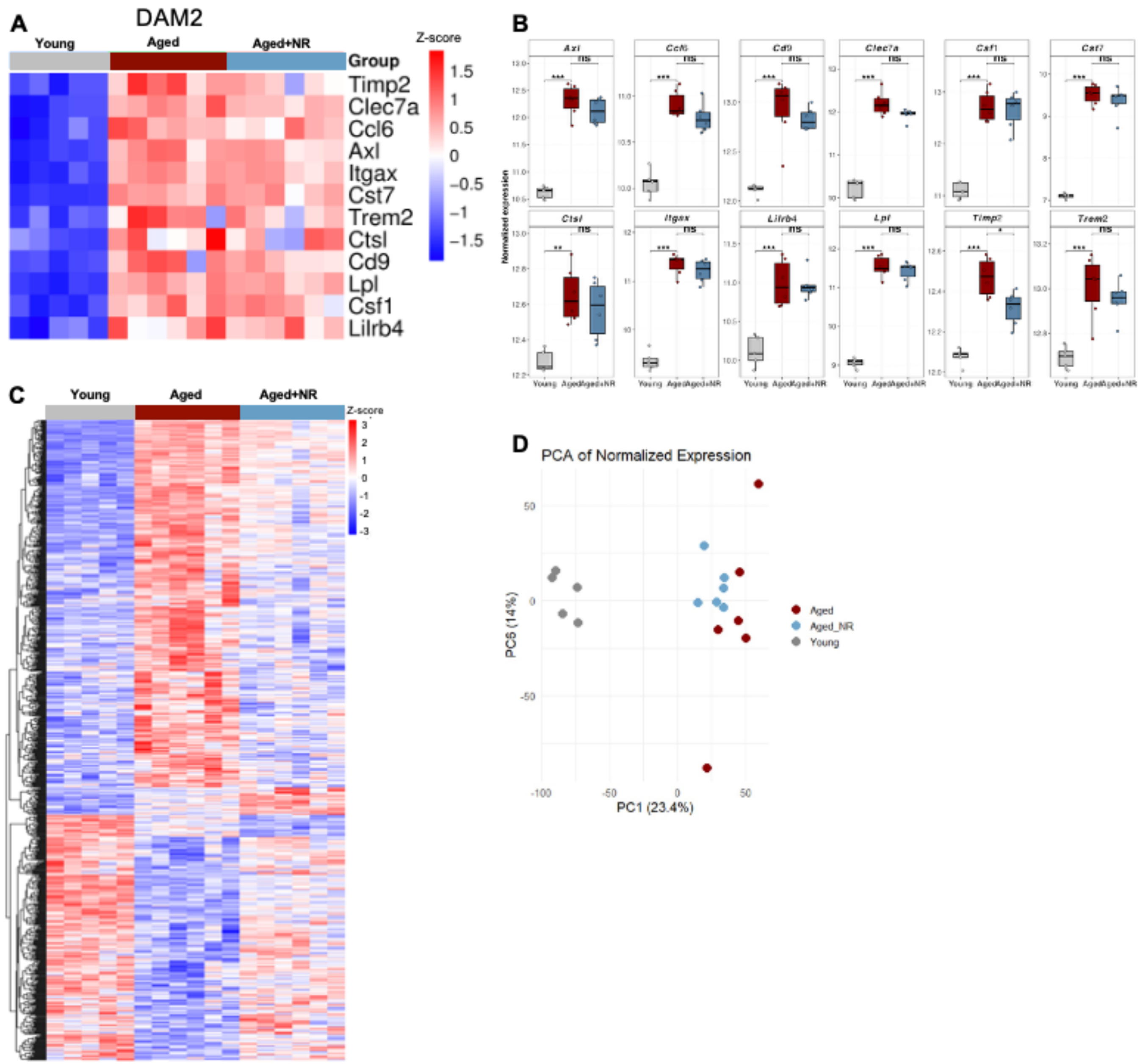

Supplementary Fig. 3: NR modulates microglial gene expression in stage 2 disease-associated phenotypes (DAM2) in aged mice. (A) Heatmap comparing gene expression profiles of homeostatic microglia in Aged and Aged+NR groups. (B) Normalized expression of DAM2-associated genes in young, aged, and aged+NR groups. (C) A heatmap displaying transcriptomic changes in microglia from Young, Aged, and Aged+NR mice. (D) Principal component analysis (PCA) of normalized expression profiles showing clear separation between Young, Aged, and Aged+NR groups, each dot represents a mouse sample. Data are presented as boxplots with individual points indicating each mouse sample.

Statistical comparisons were performed using one-way ANOVA with post hoc Tukey's test; ns = not significant, *$p < 0.05$, **$p < 0.01$, ***$p < 0.001$.

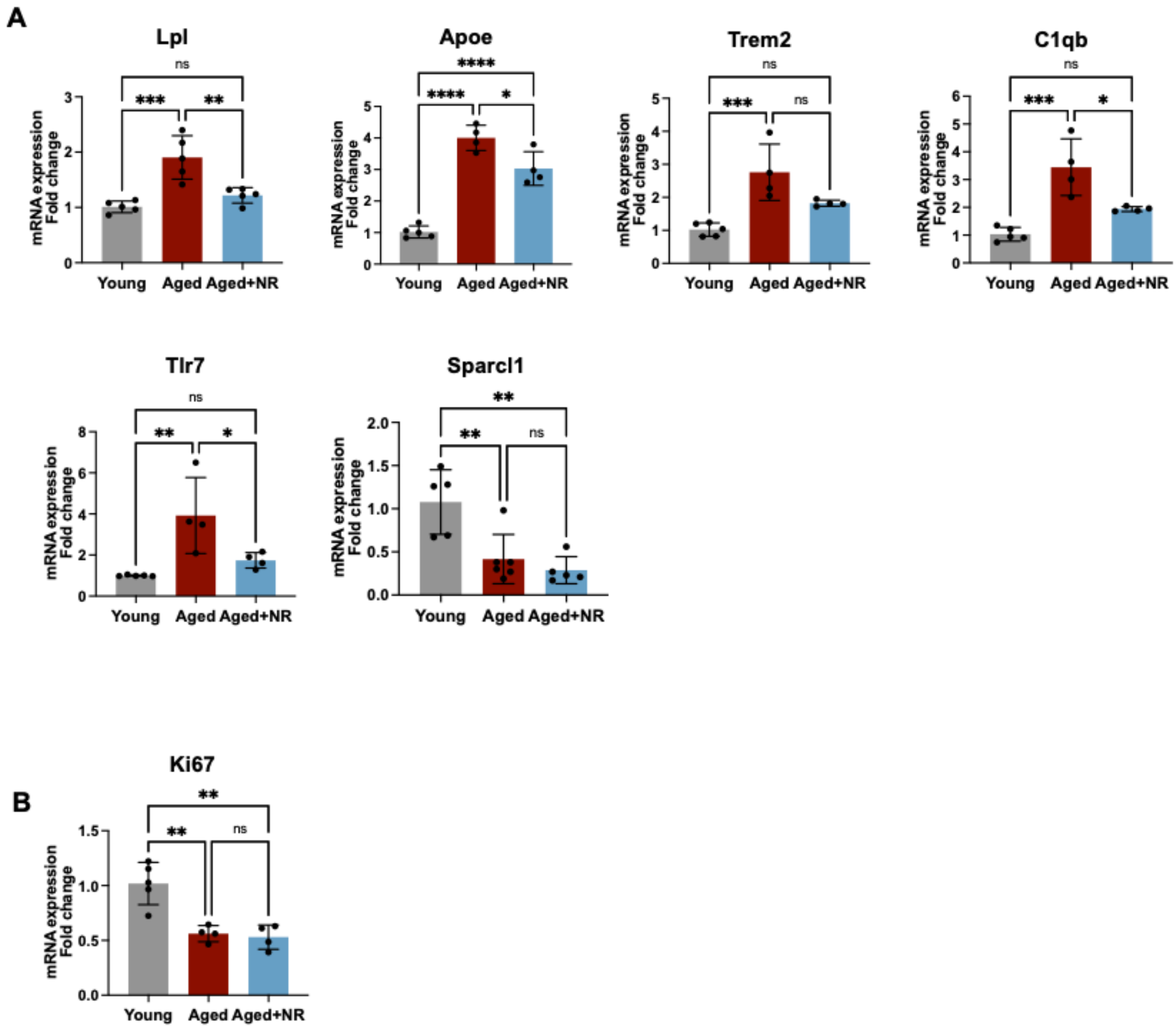

Supplementary Fig. 4: RT-qPCR validation of homeostatic microglial gene expression in young, aged, and aged+NR mice. Bar plots show relative mRNA expression of genes in microglia isolated from young, aged, and aged+NR mice. Data are presented as mean ± SDß. Statistical comparisons were performed using one-way ANOVA with post hoc Tukey's test; ns = not significant, *p < 0.05, **p < 0.01, ***p < 0.001.
